# Aerobic Exercise Facilitates the Nuclear Translocation of SREBP2 by Activating AKT/SEC24D to Contribute Cholesterol Homeostasis for Improving Cognition in APP/PS1 Mice

**DOI:** 10.3390/ijms241612847

**Published:** 2023-08-16

**Authors:** Zelin Hu, Yangqi Yuan, Zhen Tong, Meiqing Liao, Shunling Yuan, Weijia Wu, Yingzhe Tang, Yirong Wang, Changfa Tang, Wenfeng Liu

**Affiliations:** 1 Hunan Provincial Key Laboratory of Physical Fitness and Sports Rehabilitation, Hunan Normal University, Changsha 410012, China; 2 Key Laboratory of Protein Chemistry and Developmental Biology of Ministry of Education, Hunan Normal University, Changsha 410081, China

**Keywords:** Alzheimer’s disease, cholesterol, oxysterols, sterol regulatory element-binding protein-2, amyloid-beta

## Abstract

Impaired cholesterol synthesizing ability is considered a risk factor for the development of Alzheimer’s disease (AD), as evidenced by reduced levels of key proteases in the brain that mediate cholesterol synthesis; however, cholesterol deposition has been found in neurons in tangles in the brains of AD patients. Although it has been shown that statins, which inhibit cholesterol synthesis, reduce the incidence of AD, this seems paradoxical for AD patients whose cholesterol synthesizing capacity is already impaired. In this study, we aimed to investigate the effects of aerobic exercise on cholesterol metabolism in the brains of APP/PS1 mice and to reveal the mechanisms by which aerobic exercise improves cognitive function in APP/PS1 mice. Our study demonstrates that the reduction of SEC24D protein, a component of coat protein complex II (COPII), is a key factor in the reduction of cholesterol synthesis in the brain of APP/PS1 mice. 12 weeks of aerobic exercise was able to promote the recovery of SEC24D protein levels in the brain through activation of protein kinase B (AKT), which in turn promoted the expression of mem-brane-bound sterol regulatory element-binding protein 2 (SREBP2) nuclear translocation and the expression of key proteases mediating cholesterol synthesis. Simultaneous aerobic exercise restored cholesterol transport capacity in the brain of APP/PS1 mice with the ability to efflux excess cholesterol from neurons and reduced neuronal lipid rafts, thereby reducing cleavage of the APP amyloid pathway. Our study emphasizes the potential of restoring intracerebral cholesterol homeostasis as a therapeutic strategy to alleviate cognitive impairment in AD patients.

## 1. Introduction

The modified cholesterol metabolism observed in the brains of individuals with Alzheimer’s disease (AD) constitutes a significant risk factor contributing to the onset and progression of the disorder [1]. Diminished cholesterol levels have been observed within the hippocampal structures of individuals diagnosed with AD [2]. Extensive research has demonstrated that the depletion of cholesterol in the brain critically contributes to the pathogenesis of AD and is hypothesized to be closely linked to cognitive deterioration [3,4]. Intriguingly, AD patients exhibit an augmented accumulation of sterols within senile plaques [5]. Furthermore, neurons afflicted with tangles contain higher levels of unbound cholesterol compared with neighboring untangled neurons in the brains of individuals with AD [6]. However, the underlying reasons behind this phenomenon remain incompletely understood.

Maintaining cholesterol homeostasis within the central nervous system (CNS) is of paramount importance. Within the brain, cholesterol plays a crucial role in nerve growth factor signaling, facilitates the formation of lipid rafts, serves as an indispensable constituent of myelin [7,8], and constitutes a key component of synaptic vesicles, thereby sustaining synaptic activity [9]. Following neural development, cholesterol encounters restricted permeability across the blood-brain barrier (BBB) [10]. In the brain, astrocytes primarily undertake the synthesis of cholesterol [11]. Sterol regulatory element-binding protein 2 (SREBP2), a transmembrane protein, assumes a pivotal role as a transcription factor in the regulation of cholesterol synthesis [12]. SERBP2 precursor protein (pSREBP2) is a membrane protein, and when the intracellular cholesterol and oxysterol concentration is high, inactive pSREBP2 binds to SREBP cleavage-activating protein (SCAP) into pSREBP2/SCAP complexes that are anchored at the endoplasmic reticulum (ER) [13]. Conversely, when intracellular concentrations of cholesterol and oxysterols decrease, SCAP detects the alteration and dissociates from the ER, subsequently binding to the coat protein complex II (COPII). Enclosed within COPII vesicles, the pSREBP2 precursor is then transported from the ER to the Golgi complex [12,13]. Within the Golgi apparatus, a sequential cleavage process of pSREBP2 occurs through the actions of the site-1 protease (S1P) and the site-2 protease (S2P). Subsequently, mature SREBP2 (mSREBP2) is liberated and translocates to the nucleus to initiate the transcription of target genes predominantly associated with cholesterol synthesis [14,15]. Nevertheless, a reduction in the nuclear translocation of SREBP2 has been observed in individuals with AD [16].

The present study postulates that diminished nuclear translocation of SREBP2 may be correlated with the suppression of protein kinase B (AKT) activity. The accumulation of amyloid-beta (Aβ) in the brain affected by AD can impede the activation of AKT, leading to a decline in AKT activity within the AD brain [17]. Consequently, the inhibition of AKT could diminish the abundance of endogenous protein SEC24D, an integral constituent of COPII vesicles, consequently impeding the transport of COPⅡ and SREBP2 from the ER to the Golgi apparatus [18,19]. Exercise has demonstrated efficacy in activating AKT [20]. However, the specific impact of aerobic exercise on the nuclear translocation of SREBP2 in APP/PS1 mice remains an area yet to be investigated.

The underlying cause behind the formation of cholesterol deposits in the brains of individuals with AD remains elusive. Our hypothesis postulates that a significant reduction in the expression of the neuron-specific protein cytochrome P450 46A1 (CYP46A1), responsible for the extracellular transport of cholesterol in the brain, may potentially contribute to this phenomenon [21]. The elimination of excessive cholesterol within neurons is predominantly facilitated by the BBB through the enzymatic conversion of the neuron-specific enzyme CYP46A1 into the metabolite 24-S-hydroxycholesterol (24-OHC), which possesses enhanced hydrophilicity [22]. Furthermore, the accumulation of excessive intracellular cholesterol stimulates the augmentation of lipid rafts in the neuronal membrane, which is hypothesized to play a contributory role in Aβ production [23,24]. Notably, Aβ is presently acknowledged as a significant etiological factor in the cognitive decline observed in AD. Consequently, a potential detrimental feedback loop may emerge, involving the diminished presence of CYP46A1 protein within neurons, intracellular cholesterol deposition, and subsequent Aβ aggregation. However, the precise nature of this intricate relationship necessitates further elucidation through future investigations.

The neuroprotective benefits of exercise are apparent [25,26], supported by our prior research demonstrating that exercise enhances the density of dendritic spines in the neurons of APP/PS1 mice [27]. Aerobic exercise has proven to be an efficacious approach for neurodegenerative diseases. However, no pertinent investigation has been conducted to examine the impact of aerobic exercise on cholesterol synthesis in the brains of animal models with AD. Within this study, we examined the impacts of aerobic exercise on brain cholesterol synthesis in APP/PS1 transgenic mice. Our findings revealed that aerobic exercise successfully reinstated the disrupted nuclear translocation of SREBP2 and cholesterol synthesis within the brains of APP/PS1 mice. Furthermore, it restored the transportation and efflux of cholesterol in the brain while diminishing amyloid-beta precursor protein (APP) amyloid pathway cleavage. These observed effects could potentially be associated with cognitive enhancements.

## 2. Results

### 2.1. Aerobic Exercise Improves Learning Memory in APP/PS1 Mice

**Figure 1:**
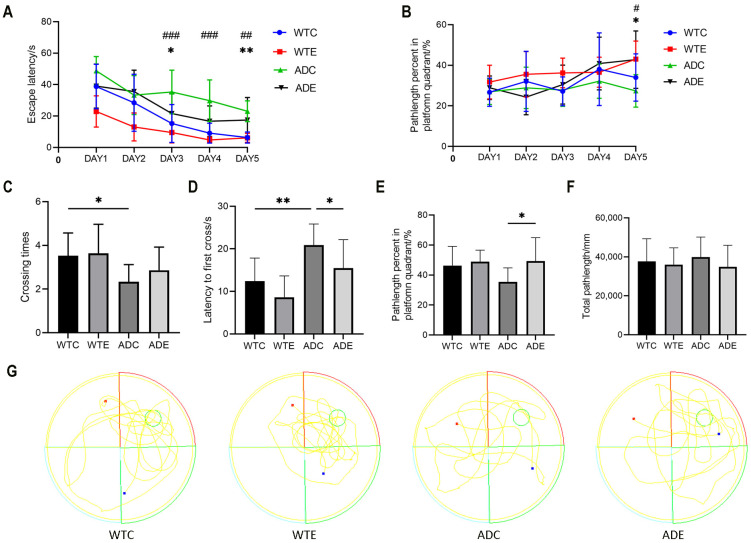
Morris water maze test (*n* = 9). (**A**) Latency changes during the Learning phase in mice. WTC group vs. ADC group; ## *p* < 0.01; ### *p* < 0.001; ADC group vs. ADE group, * *p* < 0.05; ** *p* < 0.01. (**B**) Percentage of distance travelled in the platform quadrant of the Learning phase in mice. WTE group, Day 1 vs. Day 5, # *p* < 0.05; ADE group, Day 1 vs. Day 5, * *p* < 0.05. (**C**) Number of times mice crossed the platform during the Test phase, * *p* < 0.05. (**D**) First crossing time of platform for mice in Test phase, * *p* < 0.05; ** *p* < 0.01. (**E**) Percentage of distance traveled in the platform quadrant of the Test phase mice, * *p* < 0.05. (**F**) The average swimming speed during the Test phase. (**G**) Swimming paths during the Test phase in mice. The green circle is the platform’s location. All data are shown as mean ± SD.

To assess the effect of aerobic exercise on learning and memory capacity in mice, we performed the Morris Water Maze (MWM) text. As shown in Figure 1A, aerobic exercise reduced the latency of avoidance during the learning phase in all four groups of mice; a two-way ANOVA showed that genotype (F(1,20) = 30.06, *p* < 0.0001) and aerobic exercise (F(1,20) = 23.87, *p* < 0.0001), but not the interaction (F(1,20) = 1.021, *p* = 0.4409), exerted main effects on the mouse learning phase with a main effect on avoidance latency. Post-hoc analyses showed that the avoidance latency of mice in the ADC group was significantly increased on days 3 (*p* < 0.001), 4 (*p* < 0.001), and 5 (*p* < 0.01) compared with that of the WTC group; moreover, aerobic exercise significantly reduced the avoidance latency of mice in the ADE group on days 3 (*p* < 0.05) and 5 (*p* < 0.01) compared with that of mice in the ADC group. As shown in Figure 1B, the paired-samples t-tests indicated a significant rise in the percentage of distance covered within the quadrant containing the plateau on day 5, compared with day 1, for both the WTE group of mice (*p* < 0.05) and the ADE group of mice (*p* < 0.05). As shown in Figure 1C, the number of platform traversals was reduced in APP/PS1 mice during the testing phase. A two-way ANOVA showed that only genotype had a significant main effect (F(1,20) = 7.603, *p* = 0.0095); while aerobic exercise increased the number of platform traversals in the ADE group of mice compared with the ADC group of mice, there was no significant difference. As shown in Figure 1D, a two-way ANOVA at the testing stage indicated a main effect of genotype (F(1,20) = 17.05, *p* = 0.0002) and aerobic exercise (F(1,20) = 6.119, *p* = 0.0189), but not the interaction (F(1,20) = 0.1876, *p* = 0.6679), on time to first cross the plateau. Post-hoc analyses showed that mice in the ADC group had a significant increase in time to first cross the plateau compared with the WTC group (*p* < 0.01); moreover, aerobic exercise significantly decreased the time to first cross the plateau in the ADE group compared with the ADC group mice (*p* < 0.05). As shown in Figure 1E, a two-way ANOVA during the testing phase showed that only aerobic exercise had a significant main effect (F(1,20) = 14.63, *p* = 0.0011), and that aerobic exercise increased the percentage of quadrant distance traveled by the mice’s plateau during the testing phase. Hourly swimming speed did not show significant differences between groups during the test phase (Figure 1F). Figure 1G displays the swimming trajectories of mice in each group during the testing phase. These results suggest that aerobic exercise exerts a beneficial impact on the compromised cognitive functions and memory capabilities of AD mice.

### 2.2. Aerobic Exercise Promotes Nuclear Translocation of SREBP2 in the Brains of APP/PS1 Mice

**Figure 2:**
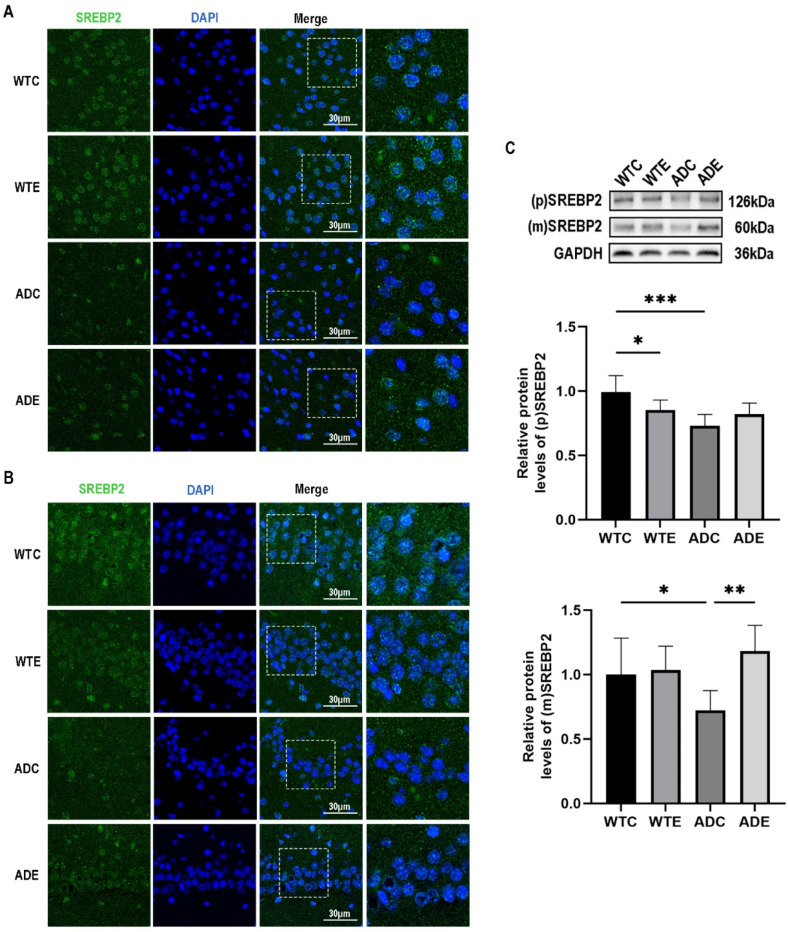
Effect of aerobic exercise on nuclear translocation of SREBP2 in mouse brain. (**A**) Immunofluorescence images of SREBP2 in mouse cerebral cortex (*n* = 3); (**B**) immunofluorescence images of SREBP2 in mouse hippocampus (*n* = 3), green fluorescence indicates SREBP2, blue fluorescence indicates nuclei, the dashed box shows the magnified portion of the rightmost image; scale bar = 30 μm. (**C**) Protein levels of pSREBP2 and mSREBP2 in mouse brain (*n* = 6). All data are shown as mean ± SD. * *p* < 0.05; ** *p* < 0.01; *** *p* < 0.001.

Studies have shown that AD patients show a reduction of SREBP2 nuclear translocation in the brain [16]. To investigate the intracellular distribution of SREBP2 in the brain of APP/PS1 mice and the effect of aerobic exercise on the nuclear translocation of SREBP2 in the brain of mice, we conducted immunofluorescence staining on tissue sections of mouse brains. As depicted in Figure 2A, a reduction in the fluorescence signal of mSREBP2 was observed in the nucleus of cells within the cortex of the ADC group when compared with the WTC group. Conversely, an enhancement in the mSREBP2 fluorescence signal was observed in the nuclei of mice in the ADE group when compared with the ADC group (Figure 2A). Similar trends were observed in the fluorescence images of the CA1 region of the hippocampus (Figure 2B). To more effectively demonstrate the effect of aerobic exercise on the nuclear translocation of SREBP2 in the mouse brain, we performed Western blot experiments for further validation. As shown in Figure 2C, a two-way ANOVA showed that genotype (F(1,20) = 20.84, *p* = 0.0002) and interaction (F(1,20) = 13.23, *p* = 0.0016), but not aerobic exercise (F(1,20) = 0.2551, *p* = 0.6191), exerted main effects on the protein content of pSREBP2 in the brain. Simple effects analysis showed that the protein content of pSREBP2 in the brain of mice in the ADC group was significantly lower (*p* < 0.001) compared with mice in the WTC group; moreover, aerobic exercise reduced the protein content of pSREBP2 in the brain of mice in the WTE group compared with mice in the WTC group (*p* < 0.05). As shown in Figure 2C, a two-way ANOVA showed that aerobic exercise (F(1,20) = 8.245, *p* = 0.0094) and interaction (F(1,20) = 6.115, *p* = 0.0225), but not genotype (F(1,20) = 0.5657, *p* = 0.4607), exerted main effects on the protein content of mSREBP2 in the brain. Simple effect analyses showed that mSREBP2 protein content in the brain of mice in the ADC group was significantly lower (*p* < 0.05) compared with mice in the WTC group; moreover, aerobic exercise significantly up-regulated mSREBP2 protein content in the brain of mice in the ADE group compared with mice in the ADC group (*p* < 0.05). The above results suggest that aerobic exercise can have a promoting effect on SREBP2 nuclear translocation in the brains of APP/PS1 mice.

### 2.3. Aerobic Exercise Activates AKT/SEC24D Signaling Pathway in APP/PS1 Mouse Brain to Promote Nuclear Translocation of SERBP2

**Figure 3:**
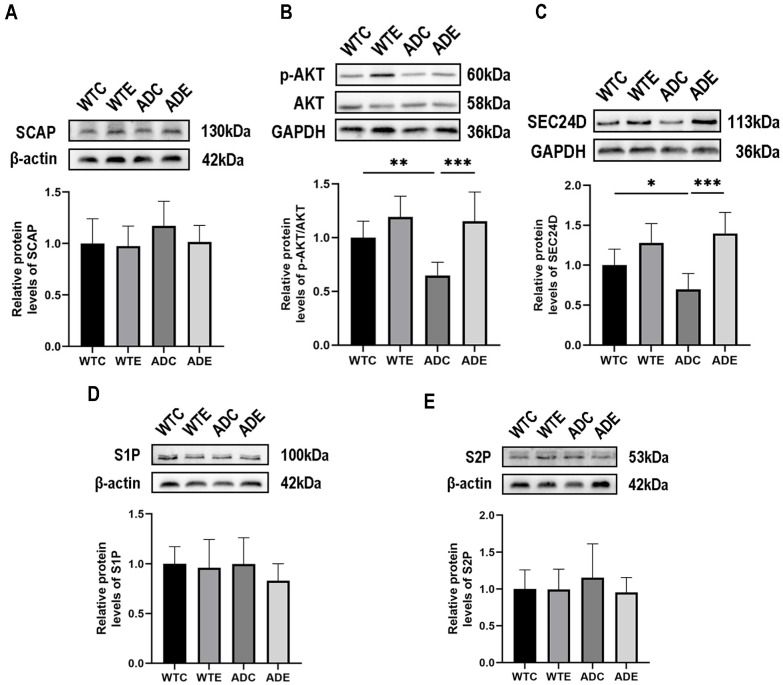
Effect of aerobic exercise on the expression of SREBP2 nuclear translocation-associated protein in mouse brain (*n* = 6). (**A**) SACP protein, (**B**) AKT and p-AKT protein, (**C**) SEC24D protein, (**D**) S1P protein, and (**E**) S2P protein. All data are shown as mean ± SD. * *p* < 0.05; ** *p* < 0.01; *** *p* < 0.001.

Since the transport of pSREBP2 from the endoplasmic reticulum to the Golgi apparatus requires the assistance of SCAP [28], we examined the content of SCAP protein in the brains of mice (Figure 3A) but found that there was no significant difference in the content of SCAP protein in the brains of mice between the groups. pSREBP2/SCAP complex transfer from the endoplasmic reticulum to the Golgi requires the assistance of COPII vesicles [12], and SEC24D, the main component of COPII vesicles, is regulated by AKT [19]. First, we examined the activation of AKT in the mouse brain. As shown in Figure 3B, two-way ANOVA showed that genotype (F(1,20) = 6.206, *p* = 0.0216) and aerobic exercise (F(1,20) = 19.72, *p* = 0.0003), but not the interaction (F(1,20) = 3.929, *p* = 0.0614), exerted main effects on the activation of AKT in the brains of the mice, and post-hoc analyses showed that the p-AKT/AKT ratio was significantly decreased in the ADC group of mice compared with the WTC group mice (*p* < 0.01); moreover, aerobic exercise significantly increased the p-AKT/AKT ratio in the brains of mice in the ADE group compared with mice in the ADC group (*p* < 0.001). This result indicated that aerobic exercise effectively improved AKT inhibition in the brains of AD mice. We next examined the protein content of SEC24D in the mouse brain. As shown in Figure 3C, a two-way ANOVA showed that aerobic exercise (F(1,20) = 6.206, *p* = 0.0216) and interaction (F(1,20) = 19.72, *p* = 0.0003), but not genotype (F(1,20) = 3.929, *p* = 0.0614), exerted main effects on the content of SEC24D in the mouse brain. Simple effect analysis showed that SEC24D protein content in the brains of mice in the ADC group was significantly lower (*p* < 0.05) compared with the WTC group; moreover, aerobic exercise significantly increased SEC24D protein content in the brains of mice in the ADE group compared with the ADC group (*p* < 0.001).

The translocation of pSREBP2/SCAP from the Golgi apparatus to the nucleus requires sequential cleavage mediated by S1P and S2P proteases, leading to the release of mature SREBP2. Consequently, we conducted an investigation into the protein levels of S1P and S2P in the mouse brain, and the findings revealed no significant differences in the expression of S1P and S2P proteins between the groups (Figure 3D,E). The above results indicate that the enhanced nuclear translocation of SREBP2 in the brains of APP/PS1 mice, facilitated by aerobic exercise, primarily arises from the activation of AKT within the brain as a consequence of aerobic exercise. Subsequently, this activation leads to an elevation in SEC24D, a significant component of COP II vesicles, ultimately resulting in the translocation of the pSREBP2/SCAP complex from the endoplasmic reticulum to the Golgi apparatus.

### 2.4. Aerobic Exercise Promotes the Expression of Cholesterol-Synthesis-Related Proteases in the Brain of APP/PS1 Mice

**Figure 4:**
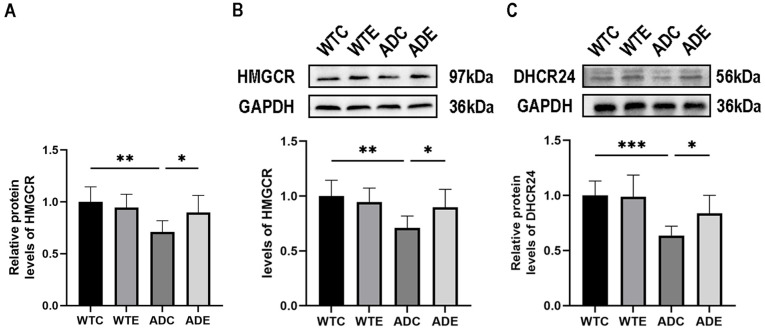
Effect of aerobic exercise on cholesterol synthesis protein in mouse brain (*n* = 6). (**A**) HMGCR mRNA, (**B**) HMGCR protein, (**C**) DHCR24 protein. All data are shown as mean ± SD. * *p* < 0.05; ** *p* < 0.01; *** *p* < 0.001.

Prior investigations have provided evidence indicating a diminished capacity for cholesterol synthesis in the brains of individuals diagnosed with AD [29], and that levels of the key proteases 3-hydroxy-3-methylglutaryl-CoA reductase (HMGCR) and 3beta-hydroxysterol-delta24 reductase (DHCR24), which mediate cholesterol synthesis, are reduced [30,31]. We first examined the gene expression of HMGCR in the mouse brain. As shown in Figure 4A, a two-way ANOVA showed that genotype (F(1,20) = 8.524, *p* = 0.0085) and aerobic exercise (F(1,20) = 7.568, *p* = 0.0123), but not the interaction (F(1,20) = 2.646, *p* = 0.1195), exerted main effects on the expression of the HMGCR gene in the mouse brain. Post-hoc analysis showed that HMGCR gene expression in the brains of mice in the ADC group was significantly decreased compared with mice in the WTC group (*p* < 0.01); moreover, aerobic exercise significantly increased HMGCR gene expression in the brains of mice in the ADE group compared with mice in the ADC group (*p* < 0.01). Secondly, we examined the protein expression of HMGCR and DHCR24 in the mouse brain. As shown in Figure 4B, a two-way ANOVA showed that genotype (F(1,20) = 9.050, *p* = 0.0069) and interaction (F(1,20) = 4.660, *p* = 0.0432), but not aerobic exercise (F(1,20) = 1.419, *p* = 0.2475), exerted the main effects on the HMGCR protein content in the mouse brain. Simple effect analysis showed that the HMGCR protein content in the brains of mice in the ADC group was significantly decreased compared with mice in the WTC group (*p* < 0.01); aerobic exercise significantly increased the HMGCR protein content in the brains of mice in the ADE group compared with mice in the ADC group (*p* < 0.05). As shown in Figure 4C, a two-way ANOVA showed that only genotype had a main effect (F(1,20) = 17.85, *p* = 0.0004). Simple effect analysis showed that DHCR24 protein content in the brains of mice in the ADC group was significantly lower compared with that in the WTC group (*p* < 0.001), and aerobic exercise significantly increased DHCR24 protein content in the brains of mice in the ADE group compared with that in the ADC group (*p* < 0.05). The above results indicated that aerobic platform running exercise contributed to the recovery of impaired cholesterol synthesis ability in the brains of AD mice.

### 2.5. Aerobic Exercise Promotes Neuronal Cholesterol Efflux and Reduces APP Amyloid Pathway Cleavage in the Brain of APP/PS1 Mice

**Figure 5:**
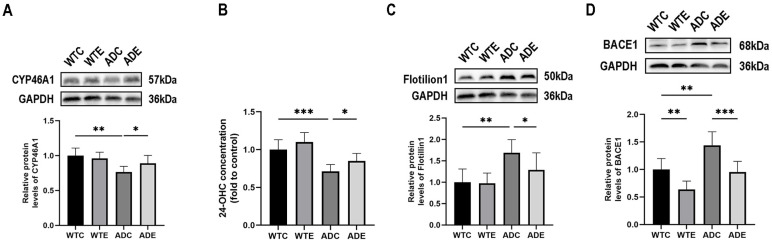
Effects of aerobic exercise on neuronal cholesterol efflux capacity, lipid rafts, and Aβ production in the mouse brain (*n* = 6). (**A**) CYP46A1 protein, (**B**) Brain 24-OHC concentration, (**C**) Flotillin1 protein, and (**D**) BACE1 protein. All data are shown as mean ± SD. * *p* < 0.05; ** *p* < 0.01; *** *p* < 0.001.

Considering the decreased level of neuron-specific cholesterol efflux protein CYP46A1 in the brains of AD patients [21] and the abnormal cholesterol deposition that occurs within neurons [6], we assessed the levels of CYP46A1, a neuronal cholesterol efflux protein, as well as its metabolite 24-OHC, in the brains of mice across each experimental group. As shown in Figure 5A, a two-way ANOVA showed that genotype (F(1,20) = 14.09, *p* = 0.0013) and interaction (F(1,20) = 4.524, *p* = 0.0461), but not aerobic exercise (F(1,20) = 1.226, *p* = 0.2814), exerted main effects on the content of CYP46A1 in the mouse brain. Simple effect analyses showed that CYP46A1 protein levels in the brains of mice in the ADC group were significantly lower (*p* < 0.01) compared with the WTC group; aerobic exercise significantly increased CYP46A1 protein levels in the brains of mice in the ADE group (*p* < 0.05) compared with the ADC group. As shown in Figure 5B, genotype (F(1,20) = 34.27, *p* < 0.0001) and aerobic exercise (F(1,20) = 6.792, *p* = 0.0169), but not the interaction (F(1,20) = 0.1662, *p* = 0.6879), exerted the main effects on 24-OHC content in the brains of the mice. Post-hoc analyses showed that 24-OHC content in the brains of mice in the ADC group was significantly decreased compared with mice in the WTC group (*p* < 0.001); aerobic exercise significantly increased 24-OHC content in the brains of mice in the ADE group compared with mice in the ADC group (*p* < 0.05).

Aβ, which is currently considered to cause cognitive impairment in AD, is mainly produced by APP in lipid rafts, and the size of lipid rafts is affected by the cholesterol concentration within neurons [32]. Flotillion1 is a representative protein marker for lipid rafts in cells, while beta-site amyloid precursor protein cleaving enzyme 1 (BACE1) is thought to be the major rate-limiting enzyme for Aβ production [33,34]. Our previous studies have shown that aerobic exercise reduces Aβ deposition in the brains of APP/PS1 mice [27]. Therefore, we further tested the effects of aerobic exercise on the levels of lipid raft markers Flotillin1 and BACE1in the brains of mice. As shown in Figure 5C, a two-way ANOVA showed that only genotype had a main effect (F(1,20) = 17.88, *p* = 0.0004) with increased Flotillion1 protein content in the brains of APP/PS1 mice. Simple effect analysis showed that Flotillion1 protein content was significantly increased in the brains of mice in the ADC group compared with the WTC group (*p* < 0.001); aerobic exercise significantly decreased Flotillion1 protein content in the brains of mice in the ADE group compared with the ADC group (*p* < 0.05). As shown in Figure 5D, a two-way ANOVA showed that genotype (F(1,20) = 21.30, *p* = 0.0002) and aerobic exercise (F(1,20) = 26.88, *p* < 0.0001), but not the interaction (F(1,20) = 0.5376, *p* = 0.4720), exerted main effects on the BACE1 protein content in the mouse brain. Post-hoc analyses showed that mice in the ADC group had significantly more BACE1 protein content in the brain compared with mice in the WTC group (*p* < 0.01) and that aerobic exercise significantly decreased BACE1 protein content in the brain of mice in the WTE group (*p* < 0.01); aerobic exercise significantly decreased BACE1 protein content in the brain of mice in the ADE group compared with mice in the ADC group (*p* < 0.001). The above results suggest that aerobic exercise reduces the content of neuronal cell membrane lipid rafts by increasing the efflux of cholesterol from neurons, which in turn reduces the cleavage of the APP amyloid pathway.

### 2.6. Aerobic Exercise Promotes Cholesterol Turnover in the Brain of APP/PS1 Mice

**Figure 6:**
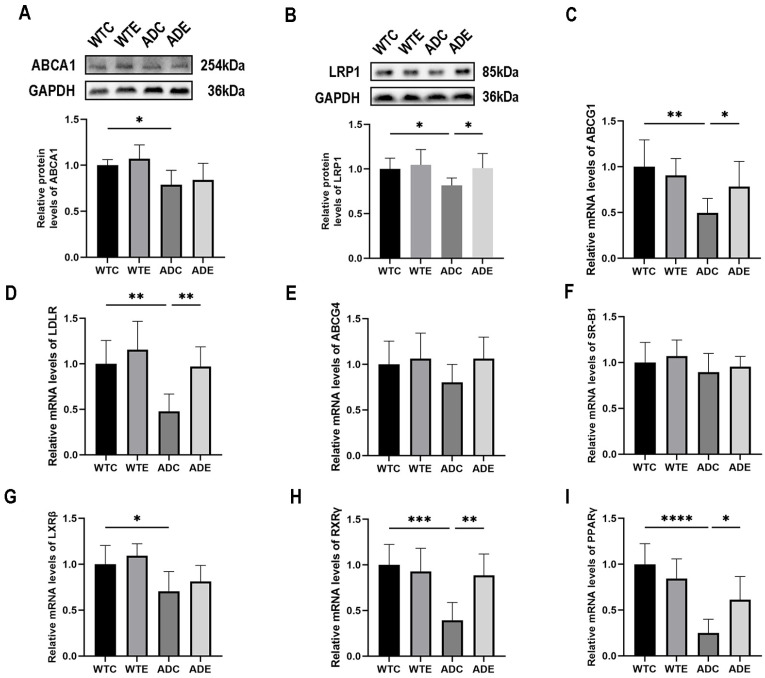
Effects of aerobic exercise on the expression of cholesterol transport proteins, genes, and related transcription factors in the mouse brain (*n* = 6). (**A**) ABCA1 protein, (**B**) LRP1 protein, (**C**) ABCG1 mRNA, (**D**) LDLR mRNA, (**E**) ABCG4 mRNA, (**F**) SR-B1 mRNA, (**G**) LXRβ mRNA, (**H**) RXRγ mRNA, and (**I**) PPARγ mRNA. All data are shown as mean ± SD. * *p* < 0.05; ** *p* < 0.01; *** *p* < 0.001; **** *p* < 0.0001.

Dysregulation of cholesterol transport is observed in the brains of individuals with AD [35]. Recent studies have demonstrated the impairment of cholesterol efflux mediation by ATP-binding cassette transporter A1 (ABCA1) and ATP-binding cassette transporter G1 (ABCG1) in the brains of individuals afflicted with AD [36]. Furthermore, a decline in the protein expression levels of low-density lipoprotein receptor-related protein 1 (LRP1), a transporter protein responsible for delivering cholesterol to neurons within the brain, has been observed [37,38]. To investigate the effects of the aerobic platform running exercise on cholesterol transport in the mouse brain, we examined the protein content of the key cholesterol transporter proteins ABCA1 and LRP1, as well as the gene expression of the key cholesterol transporter proteins ABCG1, ABCG4, LDLR, and SR-B1 in the mouse brain. As shown in Figure 6A, a two-way ANOVA showed that ABCA1 protein content was reduced in the brains of APP/PS1 mice, with only genotype having a main effect (F(1,20) = 14.08, *p* = 0.0013), whereas aerobic exercise contributed to the expression of ABCA1 protein in the brains of APP/PS1 mice, although not significantly. A two-factor ANOVA as shown in Figure 6B indicated that genotype (F(1,20) = 5.014, *p* = 0.0367) and aerobic exercise (F(1,20) = 4.695, *p* < 0.0425), but not the interaction (F(1,20) = 1.482, *p* = 0.2376), exerted main effects on the LRP1 protein content in the brains of mice. Post-hoc analysis showed that the LRP1 protein content in the brains of mice in the ADC group was significantly lower (*p* < 0.05) compared with mice in the WTC group; moreover, aerobic exercise significantly increased the LRP1 protein content in the brains of mice in the ADE group compared with mice in the ADC group (*p* < 0.05). As shown in Figure 6C, a two-way ANOVA showed that genotype (F(1,20) = 10.69, *p* = 0.0038) and interaction (F(1,20) = 5.027, *p* = 0.0364), but not aerobic exercise (F(1,20) = 1.493, *p* = 0.2360), exerted main effects on the expression of the ABCG1 gene in the mouse brain. Simple effect analysis showed that ABCG1 gene expression in the brains of mice in the ADC group was significantly lower (*p* < 0.01) compared with that in the WTC group; moreover, aerobic exercise significantly increased ABCG1 gene expression in the brains of mice in the ADE group (*p* < 0.05) compared with that in the ADC group. As shown in Figure 6D, a two-way ANOVA showed that genotype (F(1,20) = 12.15, *p* = 0.0023) and aerobic exercise (F(1,20) = 10.30, *p* = 0.0044), but not the interaction (F(1,20) = 2.795, *p* = 0.1101), exerted main effects on the expression of the LDLR gene in the brains of mice. Post-hoc analysis showed that LDLR gene expression in the brains of mice in the ADC group was significantly decreased compared with mice in the WTC group (*p* < 0.01); moreover, aerobic exercise significantly increased LDLR gene expression in the brains of mice in the ADE group compared with mice in the ADC group (*p* < 0.01). No significant differences were observed in the levels of ABCG4 and SR-B1 genes in the mouse brain between the groups (Figure 6E,F).

Furthermore, we investigated the gene expression levels of liver X receptor-beta (LXRβ), Peroxisome proliferator-activated receptor gamma (PPARγ), and retinoid X receptor gamma (RXR) RXRγ, which are transcription factors known to regulate crucial cholesterol transport [39]. As shown in Figure 6G, a two-way ANOVA showed that LXRβ gene expression was reduced in the brains of APP/PS1 mice, with only genotype having a significant main effect (F(1,20) = 14.63, *p* = 0.0011), and that aerobic exercise contributes to LXRβ gene expression in the brains of APP/PS1 mice, although not significantly. As shown in Figure 6H, a two-way ANOVA showed that genotype (F(1,20) = 12.27, *p* = 0.0022), aerobic exercise (F(1,20) = 5.171, *p* < 0.0341), and interaction (F(1,20) = 9.229, *p* = 0.0065) exerted main effects on the expression of the RXRγ gene in the mouse brain. Simple effect analyses showed that RXRγ gene expression in the brains of mice in the ADC group was significantly reduced compared with that in the WTC group (*p* < 0.001); moreover, aerobic exercise significantly increased RXRγ gene expression in the brains of mice in the ADE group compared with that in the ADC group (*p* < 0.01). As shown in Figure 6I, a two-way ANOVA showed that genotype (F(1,20) = 31.82, *p* < 0.0001) and interaction (F(1,20) = 8.890, *p* < 0.0074), but not aerobic exercise (F(1,20) = 1.422, *p* = 0.2471), exerted main effects on the expression of the ABCG1 gene in the mouse brain. Simple effect analysis showed that ABCG1 gene expression was significantly decreased in the brains of mice in the ADC group compared with the WTC group (*p* < 0.0001); moreover, aerobic exercise significantly increased ABCG1 gene expression in the brains of mice in the ADE group compared with the ADC group (*p* < 0.05). These outcomes suggest that aerobic exercise facilitates cholesterol transport in the brains of mice with AD.

### 2.7. Aerobic Exercise Improves Neurosynapses in APP/PS1 Mouse Brain

**Figure 7:**
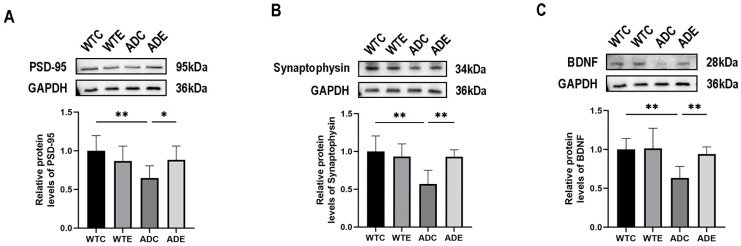
Protein expression of synapse-associated proteins and brain-derived nerve growth factor in the brain (*n* = 6). (**A**) PSD-95 protein, (**B**) Synaptophysin protein, and (**C**) BDNF protein. All data are shown as mean ± SD. * *p* < 0.05; ** *p* < 0.01.

Our previous article has shown the beneficial effects of aerobic exercise on neurons [27], and it is known that synapses are fundamental to learning and memory abilities [40]. To further evaluate the impact of cholesterol synthesis restoration in the mouse brain on neuronal function, we quantified the levels of postsynaptic density protein 95 (PSD95), synaptophysin (SYN), and brain-derived neurotrophic factor (BDNF) in mouse brain tissue. As shown in Figure 7A, a two-way ANOVA showed that genotype (F(1,20) = 5.120, *p* < 0.0349) and interaction (F(1,20) = 6.147, *p* < 0.0222), but not aerobic exercise (F(1,20) = 0.4749, *p* = 0.4987), exerted main effects on the PSD-95 protein content in the mouse brain. Simple effect analysis showed that PSD-95 protein content in the brains of mice in the ADC group was significantly lower (*p* < 0.01) compared with the WTC group; moreover, aerobic exercise significantly increased PSD-95 protein content in the brains of mice in the ADE group compared with the ADC group (*p* < 0.05). As shown in Figure 7B, a two-way ANOVA showed that genotype (F(1,20) = 9.957, *p* = 0.0050), aerobic exercise (F(1,20) = 4.572, *p* = 0.0450), and the interaction (F(1,20) = 9.874, *p* = 0.0051), exerted main effects on the SYN protein content in the mouse brain. Simple effect analyses showed that SYN protein content in the brains of mice in the ADC group was significantly lower (*p* < 0.01) compared with the WTC group; moreover, aerobic exercise significantly increased SYN protein content in the brains of mice in the ADE group compared with the ADC group (*p* < 0.01). As shown in Figure 7C, genotype (F(1,20) = 10.67, *p* = 0.0039) and aerobic exercise (F(1,20) = 4.736, *p* = 0.0417), but not the interaction (F(1,20) = 3.858, *p* = 0.0636), exerted main effects on the BDNF protein content in the mouse brain. Post-hoc analyses showed that BDNF protein content in the brains of mice in the ADC group was significantly lower (*p* < 0.01) compared with the WTC group, and aerobic exercise significantly increased BDNF protein content in the brains of mice in the ADE group compared with the ADC group (*p* < 0.01). The above results suggest that aerobic exercise contributes to the recovery of synaptic damage in the brains of APP/PS1 mice.

## 3. Discussion

Cholesterol serves as a crucial constituent of myelin and synaptic vesicles, playing an indispensable role in synaptic plasticity as well as facilitating learning and memory processes within the brain [41]. Following neurodevelopment, cholesterol is unable to traverse the BBB and is primarily synthesized by astrocytes [11]. Compelling evidence suggests impaired cholesterol synthesis within the brain of AD patients [29], and intriguingly, abnormal deposition of cholesterol within tangled neurons has been observed in individuals with AD [6]. It is well known that exercise is an effective means of preventing neurodegenerative diseases [42]. Our prior investigation has elucidated that aerobic exercise has the capacity to enhance the density of cortical and hippocampal dendritic spines while concurrently mitigating the anomalous deposition of Aβ in the brain of APP/PS1 mice [27]. In the present study, we observed that aerobic exercise ameliorated cognitive impairments in APP/PS1 mice. Furthermore, it reinstated the expression of proteins implicated in cholesterol synthesis and facilitated the nuclear translocation of SREBP2. Additionally, aerobic exercise restored the level of cholesterol transporters, thereby promoting brain cholesterol turnover. Notably, it also attenuated the cleavage of the APP amyloid pathway. These beneficial effects could potentially be attributed to the restoration of cholesterol efflux capacity in neuronal cells and the reduction of lipid raft content within the neuronal cell membrane.

The MWM test is a validated methodology for evaluating the learning and memory capabilities of mice [43]. Consequently, in order to assess the potential cognitive improvement induced by aerobic exercise in APP/PS1 mice, we initially employed the MWM experiment. The findings demonstrated that the APP/PS1 mice exhibited prolonged escape latency and fewer crossings in the platform quadrant compared with the WT mice, suggesting the presence of learning and memory impairments akin to those seen in AD. Remarkably, aerobic exercise decreased the escape latency in APP/PS1 mice and increased the number of platform crossings within the target quadrant. During the testing phase, the average swimming distance remained consistent across all groups, indicating that aerobic exercise had no impact on swimming ability. Our results provide support for the assertion that aerobic exercise holds potential for ameliorating cognitive impairments observed in individuals with AD [44].

Compelling evidence supports the presence of diminished nuclear translocation of SREBP2 within the brain affected by AD [16]. Within this study, we employed immunofluorescence imaging to visualize the nuclear translocation of SREBP2 in the CA1 region of the hippocampus and the cortex of mice. The immunofluorescence results revealed a notable reduction in SREBP2 nuclear translocation within both the cortex and hippocampus of the ADC group compared with the WTC group. Conversely, the ADE group exhibited an augmented nuclear translocation of SREBP2 in both the cortex and hippocampus compared with the ADC group, with corresponding findings observed in Western blot (WB) experiments. These findings align with the observed impairment in SREBP2 nuclear translocation detected in the brains of individuals with AD [16], thus underscoring the suitability of the APP/PS1 mouse model for investigating this phenomenon.

In cellular experiments, it was demonstrated that Aβ, a prominent pathological hallmark in the brains of both APP/PS1 mice and individuals with AD [45,46], could impede the nuclear translocation of SREBP2 via the involvement of the AKT signaling pathway [18]. Our previous study provided evidence supporting the ability of exercise to mitigate abnormal Aβ deposition in the brains of APP/PS1 mice [27]. Consequently, our hypothesis postulated that the restoration of SREBP2 nuclear translocation within the brains of APP/PS1 mice through aerobic exercise could potentially correlate with the alleviation of aberrant Aβ deposition. To comprehensively investigate this matter, we delved further into understanding the mechanisms underlying the facilitation of SREBP2 nuclear translocation by aerobic exercise. In pursuit of this goal, we assessed the protein levels of SCAP, p-AKT, AKT, SEC24D, S1P, and S2P within the mouse brain. In this study, we present compelling evidence demonstrating that aerobic exercise effectively stimulates the nuclear translocation of SREBP2. This process primarily occurs through the activation of AKT and the subsequent upregulation of its downstream effector, SEC24D, which facilitates the transportation of the SREBP2/SCAP complex to the Golgi apparatus via COPⅡ vesicles. Notably, our findings indicate that this translocation is independent of SCAP, S1P, and S2P.

Cholesterol synthesis within cells begins with the conversion of acetyl-CoA to 3-hydroxy-3-methylglutaryl coenzyme A (HMG-CoA), followed by the transformation of HMG-CoA to mevalonate catalyzed by HMGCR. Subsequently, mevalonate undergoes a series of enzymatic reactions that culminate in the production of cholesterol. The Bloch pathway involving DHCR24 holds a crucial role in mediating cholesterol synthesis in astrocytes [47]. SREBP2 exerts regulatory control over the expression of HMGCR [48]. However, a notable reduction in the expression of HMGCR and DHCR24 was observed in individuals diagnosed with AD [30,31]. Our study shows that aerobic exercise promotes cholesterol synthesis by restoring reduced levels of the proteases HMGCR and DHCR24, which mediate cholesterol synthesis, in the brains of APP/PS1 mice.

Cholesterol in neurons is mainly converted to 24-OHC by CYP46A1, which is eventually metabolized by the liver and excreted from the body [49]; this pathway represents the principal mechanism for excess cholesterol efflux in the brain [50]. Autopsy findings in individuals with AD revealed a notable decline in brain levels of CYP46A1 during the early stages, whereas a subsequent decrease in brain content of 24-OHC was observed during the later stages of AD [21]. Moreover, the accumulation of cholesterol in neurons is implicated in the generation of lipid rafts [51], and research has demonstrated that the expansion of lipid rafts enhances their interaction with APP, predominantly favoring the recruitment of β-secretase and γ-secretase enzymes crucial for APP in the amyloid metabolic pathway [52,53]. Consistently, the retention of cholesterol, coupled with augmented β-secretase and γ-secretase activity, has been found to correlate with elevated Aβ production in the brains of individuals affected by AD [24]. Evidence suggests that statins, medications aimed at lowering cholesterol levels, exhibit a potential favorable impact on the occurrence of AD; numerous studies have indicated that lipophilic statins, capable of penetrating the blood-brain barrier, reduce the risk of AD [54,55], Furthermore, certain investigations have demonstrated that employing statins to diminish the overall distribution of cholesterol in the brain may mitigate the generation and accumulation of Aβ [56]. However, in the case of AD patients, whose endogenous brain cholesterol synthesis is already compromised, the long-term efficacy of statins in inhibiting brain cholesterol synthesis appears paradoxical. This paradox is particularly notable given the age-related decline in brain cholesterol synthesis and myelination [57]. Nevertheless, this contention remains subject to ongoing debate [58]. Our study demonstrates that aerobic exercise reinstates the expression of CYP46A1 protein and restores the levels of its metabolite, 24-OHC, in the brains of APP/PS1 mice. This finding is in line with the conclusion drawn by Frank R. Sharp et al., which indicates lower levels of CYP46A1 and 24-OHC in the brains of AD mice [11]. Secondly, our results showed that aerobic exercise reduced the content of neuronal lipid rafts and BACE1 protease. This result provides a plausible explanation for the reduction of Aβ deposition in the brain of APP/PS1 mice by aerobic exercise [27,59,60]. We hypothesize that the reduction of Aβ deposition in the brains of APP/PS1 mice may be related to the restoration of CYP46A1 in the brain. This result also provides a plausible explanation for the cognitive improvement effect associated with statins.

The transport of cholesterol between astrocytes and neurons plays a crucial role in maintaining cholesterol homeostasis within the brain [61]. Astrocyte-produced cholesterol associates with apolipoprotein E (Apo E) and necessitates the assistance of ATP-binding cassette (ABC) transporter families, including ABCA1, ABCG1, and ABCG4, to facilitate cholesterol efflux from the cell [61]. Consequently, cholesterol-containing lipoproteins are internalized by neurons via specific receptors such as LDLR, LRP1, and SR-B1. Subsequently, a series of hydrolysis processes within endosomes and lysosomes leads to the release of free cholesterol (FC) [62,63]. Numerous studies have shown that alterations in the genes encoding ABCA1 and ABCG1 and abnormal ABCA1 function are associated with the risk of AD [35]. Furthermore, it has been found that by promoting the restoration of ABCA protein in the brains of APP/PS1 mice and thereby lowering cellular cholesterol levels, cognition can be improved [64]. In this study, we have presented evidence that aerobic exercise upregulates the expression of multiple cholesterol transporter genes and proteins. Previous research has indicated that aerobic exercise significantly enhances brain ABCA1 gene expression, reduces brain soluble Aβ levels, and improves cognitive function in male Wistar rats following AD induction [65], which is consistent with our experimental results.

LXR, PPAR, and RXR are nuclear transcription factors. LXR and PPAR can heterodimerize with RXR and subsequently activate the transcription of genes associated with cholesterol transport, including APOE, ABCA1, and ABCG1 [66]. In AD brains, there are observed alterations in the levels or activities of LXRβ, RXR, and PPAR [24]. It has been demonstrated that treatment with agonists for LXR and RXR leads to an increase in the levels of ABCA1 and APOE, as well as a reduction in amyloid deposition in the brains of mice overexpressing the APP [67,68]. Notably, 24-OHC, serving as a natural endogenous agonist of LXRs, has been shown to induce the up-regulation of ABCA1, ABCG1, and APOE expression [69,70]. Given the findings from the present study, which demonstrated an elevation in the brain levels of 24-OHC following exercise in APP/PS1 mice, we subsequently investigated alterations in nuclear transcription factors associated with cholesterol transport within the brain of APP/PS1 mice. The outcomes revealed that aerobic exercise resulted in an upregulation of gene expression pertaining to cholesterol transporter transcription factors. We postulated that this phenomenon may be linked to the observed increase in brain levels of 24-OHC.

Cholesterol serves as a crucial substrate for supporting neuronal growth and preserving plasticity. In our prior research, we demonstrated that aerobic exercise enhances neuronal dendritic density in mice with AD [27]. In the current study, we investigated the impact of aerobic exercise on PSD-95, SYN, and BDNF, thereby providing additional evidence for the neuroprotective effects of aerobic exercise in the brains of APP/PS1 mice.

This study has certain limitations. Our current findings reveal that aerobic exercise upregulates levels of key proteases that mediate cholesterol synthesis. However, it is important to acknowledge that cholesterol synthesis is influenced by substrate content as well. Secondly, in contrast to our findings, it has been reported that suppression of SREBP2 expression in the brains of APP/PS1 mice can diminish Aβ burden [71]. We postulate that this mechanism may be analogous to the effects observed with statins. Nevertheless, low neuronal cholesterol levels are associated with cognitive decline. It was found that cholesterol levels were reduced in the structures of the hippocampus in AD patients [72]. 6-month-old rapidly aging mouse strain senescent-accelerated mice strain 8 (SAMP8) had reduced cholesterol levels in the brain, and the loss of cholesterol is thought to be a key factor in the impaired long-term potentiation of SAMP8 mice [73]. Other investigations have demonstrated that overexpression of SREBP2 in APP/PS1 mice exacerbates neural injury [74]. Combined with our findings, we hypothesize that only when cholesterol in the brain is restored to normal levels will it contribute to the improvement of learning and memory abilities. In conclusion, our study further reveals the complex relationship between aerobic exercise, cholesterol metabolism in the brain, and cognitive function, emphasizing the potential of restoring cholesterol homeostasis in the brain as a therapeutic strategy to alleviate cognitive impairment in AD patients.

## 4. Materials and Methods

### 4.1. Experimental Animals and Grouping

Forty male mice, aged 3 months, were included in the study, comprising twenty C57BL/6J mice and twenty APP/PS1 transgenic mice. The mice were randomly assigned to four groups, with 10 mice in each group. The groups consisted of a wild-type quiet group (WTC) comprising C57BL/6J mice, a wild-type exercise group (WTE) comprising C57BL/6J mice, an AD model quiet group (ADC) comprising APP/PS1 transgenic mice, and an AD model exercise group (ADE) comprising APP/PS1 transgenic mice. C57BL/6J male wild mice and APP/PS1 male double transgenic mice were procured from Changsha, China, under license number SCXK (Xiang) 2019-0014. The ambient temperature was maintained at 23 ± 1 °C with a relative humidity between 50% and 60%. The mice were provided with sufficient food and water throughout the study. We made every effort to minimize the number of experimental animals and ensure their well-being during the course of the study.

### 4.2. Exercise Programs

Based on the preceding treadmill exercise protocols for mice [27,75], we developed the exercise protocols for mice in the WTE and ADE groups in this study. Initially, first acclimatize to running for three days, acclimatizing for 15 min per day at a pace of 5–12 m/min. Subsequently, formal training commenced in the first week at a speed of 7 m/min. The speed was gradually increased by 1 m/min every week until the eighth week, reaching a speed of 14 m/min. From the ninth week to the twelfth week, the speed was maintained at 15 m/min. Each training session lasted for 45 min, five days a week.

### 4.3. Morris Water Maze (MWM) Test

The spatial learning and memory abilities of mice were assessed using the MWM test. The test employed a circular pool with a diameter of 1.2 m and a height of 0.5 m, featuring a white interior. The pool was divided into four equal quadrants. Within the pool, a circular platform with a diameter of 10 cm was placed, and the water level was adjusted to 1.5 cm above the platform using pre-dyed white, non-toxic water-soluble dye. The water temperature was maintained at 22 °C. A video camera was positioned directly above the pool to capture the mice’s behavior during the test. The water maze test encompassed three phases: the Adaptive phase, Learning phase, and Testing phase. During the Adaptive phase, white markers were affixed to the pool’s wall above the water surface to aid in observing the mice in the water and facilitate their spatial orientation. The Adaptive phase was conducted one day prior to the Learning phase, without placing the platform. Its primary objective was to acclimate the mice to the water environment, with each mouse spending 10 min in the water. The Learning phase spanned five days, with four daily trials. Mice were randomly placed in any of the four quadrants and were tasked with locating the hidden platform within 60 s. The time taken by the mice to reach the platform was recorded as the escape latency. If a mouse successfully found the platform within the allocated time, it remained on the platform for 10 s. However, if a mouse failed to locate the platform within 60 s, it was guided to the platform and allowed to stay there for 10 s. Following the Learning phase, the platform was removed for the Testing phase. Each mouse underwent four trials, being randomly placed in the four quadrants and allowed to swim freely in the pool for 60 s. The Xeye Aba animal behavior video analysis system 3.2.5 (http://bjtmhy.bioon.com.cn/, accessed on 4 February 2023, Beijing, China) was employed for statistical analysis. Parameters such as the number of platform crossings, time spent in the platform quadrant, and total distance swum were quantified and subjected to statistical analysis.

### 4.4. Brain Tissue Collection

Following the completion of the MWM text, the mice were allowed ad libitum access to water and subjected to a 12 h fasting period. Subsequently, the mice were anesthetized with isoflurane and administered 200 mL of pre-cooled saline via cardiac perfusion. Upon decapitation, the brain tissue was expeditiously extracted on ice. For the immunofluorescence experiment, the brain tissue from three mice in each group was promptly fixed in 4% paraformaldehyde. The remaining brain tissue was rapidly submerged in liquid nitrogen and subsequently stored at −80 °C.

### 4.5. Enzyme-Linked Immunosorbent Assay

An enzyme-linked immunosorbent assay (ELISA) was conducted to quantify the concentrations of 24-OHC in the brain. The ELISA procedure was performed following the manufacturer’s instructions provided in the kit manual (SPS-20041, Saipeisenbio, Shanghai, China). Subsequently, the absorbance was measured at OD 450 nm using a spectrophotometer (BioRad, Hercules, CA, USA), and the values were correlated with standard curves.

### 4.6. Real-Time PCR

Total brain tissue RNA was extracted using TrizolTM (TRIZOLTM, Invitrogen, Carlsbad, CA), followed by qRT-PCR using the SweScript RT II First Strand cDNA Synthesis Kit (G3333-50, Servicebio, Wuhan, China). Reverse transcription was performed using 2 × SYBR Green qPCR Master Mix (G3320-01, Servicebio, Wuhan, China), and qRT-PCR reaction was performed using the CFX ConnectTM Real-Time System (Bio-Rad, Singapore). GAPDH was used as the internal reference gene, and the relative expression of the target gene was calculated by the 2^−ΔΔCT^ method. All primers used for the assay were specially designed, as detailed in Table 1.

### 4.7. Western Blotting Analysis

Approximately 30 mg of brain tissue was lysed in RIPA buffer using a low-temperature grinding instrument (Servicebio, SWE-FP, Wuhan, China). To enhance protein stability, Cocktail, PMSF, and phosphorylase inhibitors were added. The lysates were then centrifuged at 4 °C and 12,000 rpm for 15 min, followed by a 30 min incubation at 4 °C to collect the supernatant. Protein concentration was determined using the BCA protein quantification kit (Servicebio, G2026-200T, Wuhan, China). For protein analysis, the proteins were separated by acrylamide gel electrophoresis and transferred onto PVDF membranes. The membranes were subsequently blocked with 5% skim milk in TBST at room temperature for 1.5 h, followed by overnight incubation with the primary antibody at 4 °C. After incubation, the membranes were washed with TBST three times for 10 min each. Subsequently, the membranes were incubated with HRP-conjugated secondary antibodies (goat anti-rabbit IgG or goat anti-mouse IgG) at room temperature for 1.5 h. Following three washes with TBST, the PVDF membranes were immersed in an ECL developer and visualized using a Thermo Fisher imaging system. The protein bands were analyzed by optical density using ImageJ 1.51j8 software (Wayne Rasband and contributors, National Institutes of Health, Bethesda, MD, USA), with GAPDH or β-Actin serving as a normalization reference. For phosphorylated proteins, the phosphorylated proteins were incubated first, followed by incubation with the total proteins after thorough washing of the eluent. Antibodies used were as follows: SREBP2 (1:4000, 28212-1-AP, Proteintech, Wuhan, China), HMGCR (1:1000, ab242315, abcam, Cambridge, UK), DHCR24 (1:800, 10471-1-AP, Proteintech, Wuhan, China), p-AKT (1:20,000, 66444-1-Ig, Proteintech, Wuhan, China), AKT (1:20,000, 60203-2-Ig, Proteintech, Wuhan, China), SEC24D (1:2000, ab191566, abcam, Cambridge, UK), SCAP (1:1000, ab308060, abcam, Cambridge, UK), S1P (1:1000, ab140592, abcam, Cambridge, UK), S2P (1:1000, ab140594, abcam, Cambridge, UK), ABCA1 (1:1000, ab66217, abcam, Cambridge, UK), LRP1 (1:50,000, ab92544, abcam, Cambridge, UK), CYP46A1 (1:1000, 12486-1-AP, Proteintech, Wuhan, China), Flotillin1 (1:10,000, ab133497, abcam, Cambridge, UK), BACE1 (1:1000, ab183612, abcam, Cambridge, UK), Synaptophysin (1:20,000, 17785-1-AP, Proteintech, Wuhan, China), PSD-95 (1:2000, ab238135, abcam, Cambridge, UK), BDNF (1:2000, 28205-1-AP, Proteintech, Wuhan, China), GAPDH (1:2000, GB15004, servicebio, Wuhan, China), β-Actin (1:2000, GB15003, servicebio, Wuhan, China), HRP conjugated Goat Anti-Mouse IgG (1:5000, GB23301, servicebio, Wuhan, China), and HRP conjugated Goat Anti-Rabbit IgG (1:3000, GB23303, servicebio, Wuhan, China).

### 4.8. Immunofluorescence Staining

Paraffin-embedded mouse brain tissues were sectioned coronally at a thickness of 5 μm. After deparaffinization, the sections underwent antigen retrieval using an antigen repair solution (Servicebio, Cat: G1207, Wuhan, China) following the provided instructions. Once the repair process was completed, the sections were allowed to naturally cool and then washed three times with PBS (pH 7.4) for 5 min each. Bovine serum albumin (BSA) was applied to the cells and incubated at room temperature for 30 min. Primary antibodies were then added to the sections and left to incubate overnight at 4 °C. After the incubation period, the sections were washed three times with PBS (pH 7.4) for 5 min each. Subsequently, the corresponding HRP-labeled secondary antibody was added and incubated at room temperature for 50 min. Following another round of washing with PBS (pH 7.4) three times for 5 min each, the sections were incubated with CY3-TSA (Servicebio, Cat: G1223, Wuhan, China) in the dark at room temperature for 10 min. After incubation, the slides were placed in TBST and washed three times on a decolorization shaker for 5 min each. DAPI staining solution (Servicebio, Cat: G1012, Wuhan, China) was applied to the sections and incubated at room temperature in the dark for 10 min. The sections were then washed three times with PBS (pH 7.4) for 5 min each. To quench autofluorescence, an autofluorescence quenching agent was applied for 5 min, followed by rinsing with running water for 10 min. Finally, the sections were sealed, and images were captured using an upright fluorescence microscope (Nikon Eclipse C1, Nikon, Tokyo, Japan).

### 4.9. Statistical Analysis

Statistical analysis and data visualization were conducted using GraphPad Prism 9.0.0 software (GraphPad Software, San Diego, CA, USA). A two-way ANOVA was performed to compare the effects of genotype and exercise on mice, and if one or both factors produced a main effect and there was no interaction, post-hoc multiple comparisons were performed using the LSD method to assess the statistical significance of differences between groups. If a factor did not produce a main effect or there was an interaction, a simple effects analysis was further conducted using an independent sample *t*-test to determine the effect of this factor within the group. A paired-sample *t*-test (two-tailed) was used to compare the difference in the percentage of distance traveled in the plateau quadrant within the group on Day 1 vs. Day 5 of MWM text. The data were presented as mean ± standard deviation (mean ± SD). *p* < 0.05 was considered statistically significant.

## Figures and Tables

**Table 1:** Sequences of primers for target genes.

Gene	Forward Primer	Reverse Primer
*LXRβ*	CTGAAGGCGTCCACCATTGAGATC	TGATGGCGATAAGCAAGGCATACTC
*PPARγ*	GCCAAGGTGCTCCAGAAGATGAC	GTGAAGGCTCATGTCTGTCTCTGTC
*RXRγ*	GGAGCCGAGAGCGAGCAGAG	CCACGTTCATGTCACCGTAGGATTC
*LDLR*	TGAGGTTCCTGTCCATCTTCTTCC	GATGTTCTTCAGCCGCCAGTTC
*SR-B1*	GTGCCCATCATCTGCCAACTG	GCTGTCCGCTGAGAGAGTCC
*ABCG1*	TGCTGCTGCCTCACCTCAC	TCTCGTCTGCCTTCATCCTTCTC
*ABCG4*	ACATGCTACTGCCTCACCTCAC	GTTCCTTCTTCACCTCTTGCTTCTC
*GAPDH*	TGAAGGTCGGTGTGAACGGATTTG	TCGCTCCTGGAAGATGGTGATGG

## Data Availability

Data described in the manuscript are available from the corresponding author on reasonable request.
